# Salivary cathelicidin LL-37 as a non-invasive biomarker for oral cancer detection

**DOI:** 10.6026/973206300223421

**Published:** 2026-06-30

**Authors:** Thaneja Ravichandran, Harshavardhan B.G, Nandhini Ganesan, Sarswathi Gopal K

**Affiliations:** 1 Department of Oral Medicine and Radiology, Meenakshi Ammal Dental College and Hospital, Meenakshi academy of higher education and research, Chennai, Tamil Nadu, India

**Keywords:** Biomarker, oral squamous cell carcinoma (OSCC), oral potentially malignant disorder (OPMD), oral malignancy

## Abstract

Oral squamous cell carcinoma (OSCC) is the predominant form of oral malignancy, with late-stage diagnosis contributing to poor prognosis. Oral potentially malignant disorders (OPMDs) often precede OSCC, underscoring the need for early diagnostic biomarkers. Hence, salivary cathelicidin LL-37 levels were quantified using ELISA in 80 participants. Participants were divided into four groups: OSCC, tobacco- associated OPMDs, non-tobacco-associated OPMDs, and healthy controls. Salivary cathelicidin LL-37 levels increased progressively from healthy controls to OSCC cases (p < 0.001) with 100% sensitivity and 90% specificity. Thus, we show that salivary LL-37 is a reliable, non-invasive biomarker for early detection and monitoring of oral carcinogenesis.

## Background:

Oral squamous cell carcinoma accounts for nearly 90% of all oral cancers and represents a significant global health burden, particularly where tobacco and alcohol exposure is prevalent [1]. The prognosis remains poor due to delayed diagnosis at advanced stages [2]. Early detection significantly improves survival rates; however, current diagnostic methods are often invasive, expensive, or not feasible for large-scale screening [3]. OPMDs, including leukoplakia, oral submucous fibrosis and oral lichen planus, are recognized OSCC precursors and provide an opportunity for early intervention [4]. Saliva has gained increasing attention as a diagnostic biofluid due to its ease of collection, non-invasive nature and ability to reflect local and systemic physiological changes [5]. It contains proteins, cytokines, enzymes and nucleic acids investigated as potential biomarkers for oral diseases [6]. Antimicrobial peptides are essential components of innate immunity, with functions extending beyond microbial defense to include modulation of inflammation, wound healing and immune responses [7]. Cathelicidin LL-37 is the only known human cathelicidin, derived from the precursor protein hCAP18 [8]. LL-37 plays a dual role in cancer biology, acting as either a promoter or inhibitor of tumor progression depending on cellular context and the tumor microenvironment [9]. Elevated expression has been reported in breast and lung cancers, while reduced expression has been noted in others [10]. In the oral cavity, LL-37 is produced by epithelial and immune cells in response to inflammatory stimuli [11]. Chronic inflammation a key driver of oral carcinogenesis may influence its expression levels progressively across stages of oral disease. Therefore, it is of interest to report the role of salivary proteins, cytokines, enzymes and nucleic acids as potential biomarkers for oral diseases.

## Methodology:

A prospective cross-sectional study was conducted involving 80 participants divided equally into four groups: OSCC, tobacco-associated OPMDs, non-tobacco-associated OPMDs and healthy controls (n = 20 each). Participants were recruited following clinical diagnosis and written informed consent. Inclusion criteria comprised clinically diagnosed OSCC and OPMDs (leukoplakia, oral submucous fibrosis, oral lichen planus). Individuals with systemic diseases, prior malignancy, ongoing treatment, or conditions affecting salivary flow were excluded. Unstimulated whole saliva (3-5 mL) was collected under standardized conditions after a minimum one-hour fast. Samples were centrifuged to remove debris; supernatants were stored at -80°C until analysis. Salivary LL-37 levels were quantified by sandwich ELISA (optical density measured at 450 nm). Statistical analysis used SPSS v27.0; data normality and homogeneity were verified before one-way ANOVA with Tukey post-hoc test. A p-value < 0.05 was considered statistically significant.

## Results:

The socio-demographic characteristics of the study participants are presented in Table 1. A total of 80 participants were included and divided equally into four groups: oral squamous cell carcinoma (OSCC), tobacco-associated oral potentially malignant disorders (OPMDs), non-tobacco-associated OPMDs, and healthy controls. The mean age was highest among OSCC patients (54.7 ± 11.75 years) and lowest among healthy controls (35.15 ± 13.35 years). Male predominance was observed in the OSCC and tobacco-associated OPMD groups, whereas females constituted the majority of the control group. The comparison of salivary cathelicidin LL-37 levels among the four study groups is shown in Table 2. Mean salivary LL-37 levels demonstrated a progressive increase from healthy controls to patients with OSCC. The highest mean level was observed in the OSCC group (6.62 ± 0.30), followed by the tobacco-associated OPMD group (5.38 ± 0.30), the non-tobacco-associated OPMD group (4.17 ± 0.10), and the healthy control group (3.22 ± 0.40). One-way ANOVA revealed a statistically significant difference in salivary LL-37 levels among the study groups (p < 0.001). Post-hoc Tukey analysis (Table 3) demonstrated statistically significant differences between all pairwise group comparisons (p < 0.001). The greatest mean difference was observed between the OSCC and healthy control groups (mean difference = 3.40), indicating substantially elevated LL-37 levels in OSCC patients. Significant differences were also observed between OSCC and tobacco-associated OPMDs (mean difference = 1.24), OSCC and non-tobacco-associated OPMDs (mean difference = 2.44), tobacco-associated OPMDs and controls (mean difference = 2.15), and non-tobacco-associated OPMDs and controls (mean difference = 0.95). Receiver operating characteristic (ROC) curve analysis was performed to evaluate the diagnostic performance of salivary LL-37 levels in differentiating diseased individuals from healthy controls. As illustrated in Figure 1 (see PDF), salivary LL-37 demonstrated excellent diagnostic accuracy, with high sensitivity and specificity for distinguishing pathological lesions from healthy individuals. The ROC curve showed an area under the curve indicative of strong discriminatory ability, supporting the potential utility of salivary LL-37 as a non-invasive biomarker for the early detection and monitoring of oral potentially malignant disorders and oral squamous cell carcinoma.

## Discussion:

This study demonstrates a statistically significant progressive increase in salivary LL-37 levels from healthy controls to OPMDs and OSCC, with the highest levels in OSCC patients followed by tobacco-associated and non-tobacco-associated OPMDs. This pattern is consistent with cumulative inflammatory and molecular changes underlying oral carcinogenesis. Chronic inflammation promotes genetic instability, cellular proliferation and tissue remodeling in cancer development [12]. As an immunomodulatory peptide, LL-37 is upregulated in response to inflammatory stimuli, supporting its utility as an indicator of ongoing pathological processes. Higher LL-37 levels in tobacco-associated OPMDs compared to non-tobacco lesions support the role of environmental carcinogens in amplifying inflammatory responses [13]. Similar findings were reported by Tarrad *et al.* who demonstrated significantly elevated salivary LL-37 levels in patients with oral potentially malignant disorders compared with healthy controls, highlighting its potential role as an early biomarker of oral carcinogenesis [14]. Tobacco exposure induces oxidative stress and cytokine release, which may enhance LL-37 expression. The diagnostic performance of LL-37 with high sensitivity and specificity is consistent with findings for other salivary biomarkers such as IL-6 and TNF-α in oral cancer [15], reinforcing the concept of saliva-based diagnostics While reduced tissue expression of LL-37 has been reported in advanced cancers due to epigenetic alterations, elevated salivary levels may reflect a combined response from epithelial cells, immune cells and the tumor microenvironment [14]. This underscores the distinction between tissue and salivary LL-37 expression profiles.

## Conclusion:

Salivary LL-37 levels increase progressively with oral mucosal disease severity, reflecting inflammation and immune activation in oral carcinogenesis. Its high diagnostic performance and non-invasive collection support its potential as a biomarker for early detection and disease monitoring. Large-scale longitudinal studies are warranted to validate these findings and establish standardized clinical protocols.

## Figures and Tables

**Table 1:** Socio-demographic details of study participants

		**Group 1 (OSCC)**	**Group 2 (Tobacco OPMDs)**	**Group 3-4**
Age	M±SD	54.7±11.75	50.20±11.75	See below
Gender n (%)	Males	20 (100)15 (83.3)	-	
	Females	0(0)	3(16.7)	-
Note: Group 3 (Non-tobacco OPMDs):
Age 42.3±13.11;
Males 11 (55%), Females 9 (45%).
Group 4 (Controls): Age 35.15±13.35;
Males 5 (25%), Females 15 (75%).

**Table 2:** Comparison of salivary cathelicidin levels between study groups

	**Mean**	**SD**	**95% CI Lower**	**95% CIUpper**	**p-value**
Group 1 (OSCC)	6.62	0.3	6.48	6.756	<0.001*
Group 2 (Tobacco OPMDs)	5.38	0.3	5.25	5.5	
Group 3 (Non-tobacco OPMDs)	4.17	0.1	4.14	4.21	
Group 4 (Controls)	3.22	0.4	3.03	3.4	
One-way ANOVA; *p <
0.05 statistically significant

**Table 3:** Post-hoc comparisons of salivary cathelicidin levels between study groups

**Comparisons**	**Mean Difference**	**p-value**
Group 1 vs Group 2	1.24	<0.001*
Group 1 vs Group 3	2.44	<0.001*
Group 1 vs Group 4	3.4	<0.001*
Group 2 vs Group 3	1.2	<0.001*
Group 2 vs Group 4	2.15	<0.001*
Group 3 vs Group 4	0.95	<0.001*
Post-hoc Tukey test;
*p < 0.05 statistically significant
